# Supplementary material for: Scutellarein Suppresses the Production of ROS and Inflammatory Mediators of LPS-Activated Bronchial Epithelial Cells and Attenuates Acute Lung Injury in Mice
Source: Antioxidants (Basel). 2024 Jun 12;13(6):710. doi: 10.3390/antiox13060710 (PMC11200809; doi:10.3390/antiox13060710)
Supplement: Supplementary file 1 [file antioxidants-13-00710-s001.zip › antioxidants-3020277-supplementary.pdf]

Methods

1. Superoxide anion scavenging activity assay

Superoxide anion scavenging activity of scutellarein was determined as previously described with slight modifications [52]. In a 96-well plate, 50 μL of serial dilutions of scutellarein, 50 μL of PMS (150 μM), 50 μL NADHNa$_2$ (1.2 mM) were mixed thoroughly. After adding 50 μL of NBT (360 μM), the reaction was initiated and incubated at room temperature for 5 min, and the optical density at 540$_{nm}$ was measured.

2. Total anti-oxidative capacity assay

Total anti-oxidative capacity of scutellarein was determined as previously described with slight modifications [53]. In a 96-well plate, the TPTZ reagent was prepared by mixing TPTZ (10 mM), acetate buffer saline (0.3 M, pH 3.6), and ferric chloride (FeCl3, 20 mM) in a 10:1:1 ratio. For the assay, 200 μL of the reaction mixture, including 100 μL of TPTZ reagent and serial dilutions of scutellarein, was incubated at 37°C for 20 min. The optical density was then measured at 540nm. The total antioxidative capacity of scutellarein was quantified as Trolox-equivalent antioxidant capacity.

3. DPPH radical scavenging activity assay

DPPH radical scavenging activity of scutellarein was assayed as previously described [54]. In a 96-well plate, an anhydrous ethanol DPPH solution (0.5 mM) was mixed with serial dilutions of scutellarein. The mixture was then incubated at room temperature for 20 min and the optical density at 540$_{nm}$ was measured.

4. Hydroxyl radical scavenging activity assay

Hydroxyl radical scavenging activity of scutellarein was assessed by the method as previously described [55]. In a test tube, the following solutions were sequentially added and mixed thoroughly: 50 μL of EDTA (2 mM), 50 μL of FeCl$_3$ (2 mM), 50 μL of H_2O_2 (20 mM), 180 μL of deoxyribose (2.8 mM), 455 μL of scutellarein, 165 μL of phosphate buffer (0.1 M, pH 7.4), and 50 μL of VC (2 mM). After incubating in a water bath for 60 min, 1 mL of TCA (10%) and TBA (67 mM), along with 10 μL of anhydrous ethanol BHT solution (54.46 mM) were introduced. The mixture was then heated in a boiling water bath for 30 min. Subsequently, 2 mL of n-butanol was added for vortex extraction. The tube was centrifuged at 1000 *g* for 10 min, and 200 μL of the supernatant was transferred to a 96-well black plate. The fluorescence intensity was then read at λ_{ex} 510$_{nm}$ / λ_{em} 590$_{nm}$ by a fluorescence microplate analyzer (Fluoroskan Ascent FL, Thermo Fisher Scientific, USA).

5. Anti-lipid peroxidation assay

The anti-lipid peroxidation assay was conducted as previously described with slight modifications [56]. Briefly, fresh rat liver was homogenized on ice with 0.15 M KCl solution using a tissue homogenizer to prepare a 20% (w/v) liver homogenate. The homogenate was then centrifuged at 2500 *g* for 10 min at 4°C, and 100 μL of the supernatant was added into the test tube, followed by 50 μL of serially diluted aqueous solution of scutellarein and 100 μL of $FeSO_4$ (3 mM). The mixtures were incubated at 37°C for 60 min. Subsequently, 20 μL of BHT (90 mM) was added and mixed thoroughly. Then, 1 mL of TBA (50 mM) and 0.5 mL of TCA (5.6%) were added, mixed, and heated in a boiling water bath for 30 min. Subsequently, 1.5 mL of n-butanol was added for vortex extraction. After centrifugation at 1000 *g* for 10 min, 300 μL of the supernatant was transferred to a 96-well black plate. The fluorescence intensity was then read at λ_{ex} 510$_{nm}$ / λ_{em} 590$_{nm}$ by a fluorescence microplate analyzer (Fluoroskan Ascent FL, Thermo Fisher Scientific, USA). The MDA content was calculated using the standard curve of TEP.

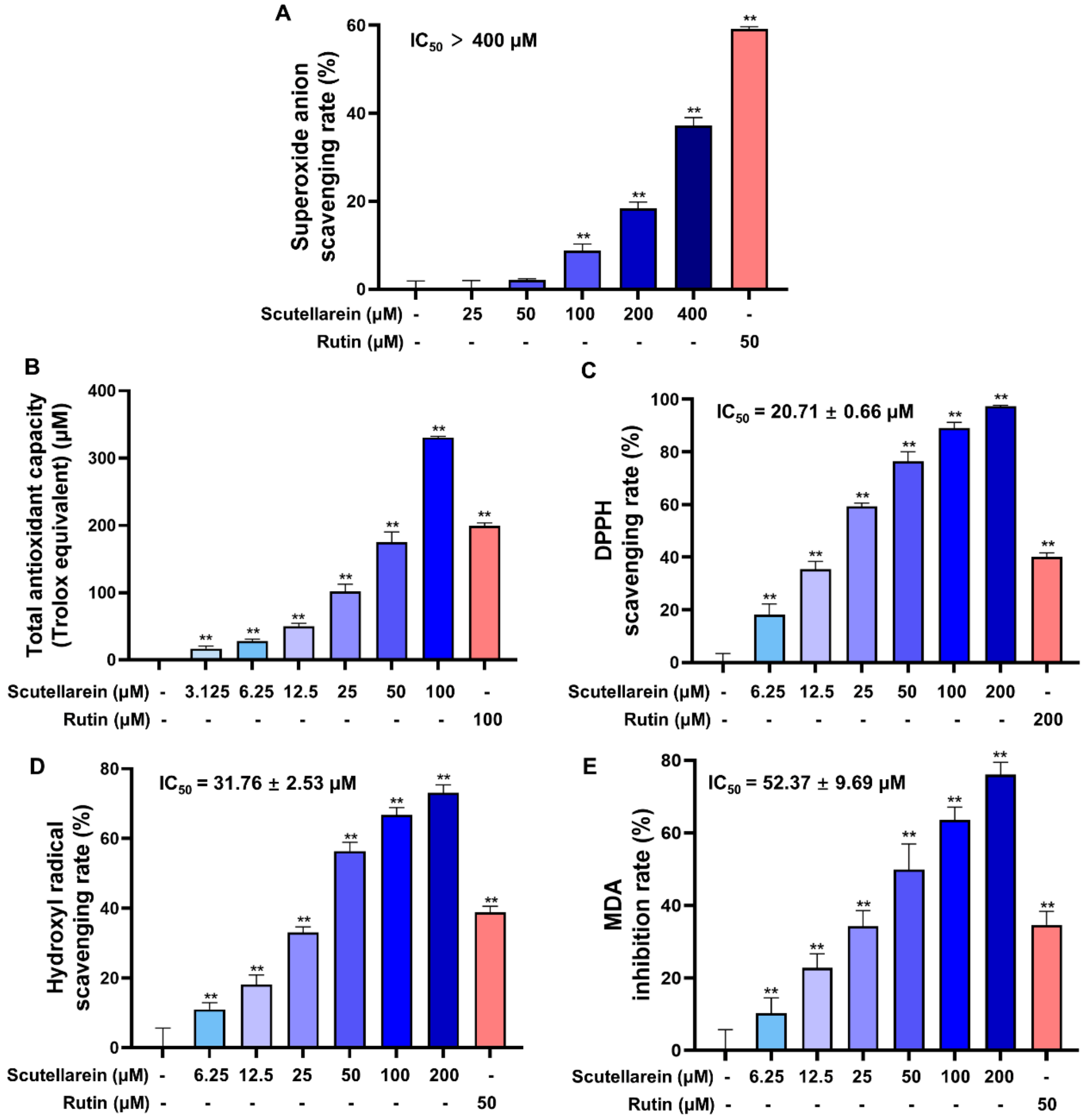

Figure S1. The anti-oxidative activity of scutellarein in cell-free systems (n = 3). (**A**) Superoxide anion scavenging activity of scutellarein. (**B**) Ferric reducing ability of scutellarein. (**C**) DPPH radical scavenging activity of scutellarein. (**D**) Hydroxyl radical scavenging activity of scutellarein. (**E**) Effect of scutellarein on Fe^{2+}-induced MDA formation. Rutin served as a positive control. $^{**}P < 0.01$ *vs.* negative control.
